# Current Evidence on the Bioavailability of Food Bioactive Peptides

**DOI:** 10.3390/molecules25194479

**Published:** 2020-09-29

**Authors:** Lourdes Amigo, Blanca Hernández-Ledesma

**Affiliations:** Department of Bioactivity and Food Analysis, Institute of Research in Food Sciences (CIAL, CSIC-UAM, CEI-UAM+CSIC), Nicolás Cabrera 9, 28049 Madrid, Spain; lourdes.amigo@csic.es

**Keywords:** digestion, absorption, food peptides, bioavailability, biological activity, encapsulation

## Abstract

Food protein-derived bioactive peptides are recognized as valuable ingredients of functional foods and/or nutraceuticals to promote health and reduce the risk of chronic diseases. However, although peptides have been demonstrated to exert multiple benefits by biochemical assays, cell culture, and animal models, the ability to translate the new findings into practical or commercial uses remains delayed. This fact is mainly due to the lack of correlation of in vitro findings with in vivo functions of peptides because of their low bioavailability. Once ingested, peptides need to resist the action of digestive enzymes during their transit through the gastrointestinal tract and cross the intestinal epithelial barrier to reach the target organs in an intact and active form to exert their health-promoting properties. Thus, for a better understanding of the in vivo physiological effects of food bioactive peptides, extensive research studies on their gastrointestinal stability and transport are needed. This review summarizes the most current evidence on those factors affecting the digestive and absorptive processes of food bioactive peptides, the recently designed models mimicking the gastrointestinal environment, as well as the novel strategies developed and currently applied to enhance the absorption and bioavailability of peptides.

## 1. Introduction

In the last few years, food-derived bioactive peptides have attracted the interest of scientists because of their safety, low cost, and benefits on health beyond the nutritive role. Bioactive peptides have been demonstrated to positively affect the major body systems, notably, the cardiovascular, digestive, endocrine, immune, and nervous systems, while minimizing the risks of chronic disease development [1]. Thus, they have become promising ingredients for functional foods and nutraceuticals [2,3]. A lot of in vitro biochemical assays, cell models, and animal models have been optimized and applied for testing the bioactivity of these food bioactive peptides. However, although the research on the development of peptides-enriched products has notably increased, the ability to translate the new findings into practical or commercial uses remains delayed. Among the major reasons behind this delay, one of the most important is the lack of correlation of the in vitro bioactivities of peptides with in vivo functions due to their low bioavailability following oral administration [4]. Peptides need to resist the action of digestive enzymes during their transit through the gastrointestinal tract and cross the intestinal epithelial barrier to reach intact the target organs where peptides can exert their health-promoting effects [5]. Thus, when studying the effects of bioactive peptides in our organism, it is important to assess their under-digestive conditions, and if the peptide is absorbed, it is necessary to evaluate its distribution, metabolism, and excretion behavior [6,7]. In this review, the most current evidence on the in vitro and in vivo models designed to evaluate the digestibility and bioavailability of food bioactive peptides is summarized, focusing on those limiting factors affecting both peptides resistance to digestive conditions and absorption capacity.

## 2. Bioavailability of Food Peptides

### 2.1. Digestibility of Food Peptides

Human digestion is a complex process that involves the concerted action of digestive enzymes on dietary ingredients. In the case of digestion of food proteins, several factors influence this process such as the type of proteins, gastric and intestinal pH, activity of digestive enzymes, endogenous secretions, and motility [8]. Digestion is considered a vital process for life because nutrients released from ingested foods are used by the body as an energy source for cell maintenance and growth [9]. During the digestion of food proteins, peptides and amino acids are liberated, acting as signals of gastric or intestinal motility and pancreatic secretion, and/or exerting local and systemic physiological functions.

Digestion starts with a short food chewing step in the mouth, which is relevant for the complete digestive process, particularly for the gastric emptying rate [10]. The food bolus resulting from mechanical and enzymatic degradations in the mouth is transported through the esophagus to the stomach by peristaltic movements. Once the bolus reaches the proximal part of the stomach, it is mixed with the gastric juice, which is mostly composed of hydrochloric acid (HCl), pepsin and lipases responsible for protein and lipid digestion, respectively, and mucus that protects the mucosal surface. In the distal part, peristaltic movements allow breaking large food particles into smaller ones by grinding and mixing gastric contents. The stomach ends at the pylorus that pumps small particles (chyme) to the duodenum, while the largest particles are maintained in the stomach for further digestion. Once the chyme enters the duodenum, its acidic pH is neutralized by sodium carbonate (NaHCO_3_) until reaching a pH appropriate for the activity of pancreatic (proteases, amylases, and lipases) and intestinal enzymes, which are responsible for the subsequent digestion of molecules contained in the chyme. Bile produced by the liver contributes to lipid digestion by emulsifying dietary fats into small droplets that favor the activity of lipase. Once digested, released nutrients are available for their absorption by villus enterocytes through different transport mechanisms, and non-absorbed material travels down to the large intestine. In the colon, water and electrolytes are absorbed, bile salts are reabsorbed, and non-digested polysaccharides and proteins are fermented by colonic microbiota, releasing new degradation products. Finally, at the end of the large intestine, the formation, storage, and elimination of feces occurs [11].

#### 2.1.1. Parameters Limiting Peptides Digestibility

The digestive processes, including gastric emptying, intestinal transit, secretion of digestive fluids and mucus, and motility are influenced by many factors such as physical and chemical characteristics, food composition, and physiological factors [12,13]. Among physicochemical factors, low pH and temperature, high osmolality, viscosity, fiber content, and energy density (caloric content) have been demonstrated to delay gastric emptying, while food volume increases the gastric emptying rate. In addition, the particle size and the degree of hydrolysis of meal constituents may also play a significant role [14].

Regarding the type of food constituent, and specifically for proteins, the site of digestion varies depending on their food source. As an example, while bovine casein has been found to precipitate in the stomach where it is hydrolyzed, soluble whey and soybean proteins pass rapidly through the stomach, reaching the duodenum where are hydrolyzed by pancreatic proteases [15]. This different behavior of proteins under gastrointestinal digestion determines the subsequent absorption of released peptides. Pepsin cleaves peptic bonds next to aromatic amino acids such as phenylalanine, tryptophan, and tyrosine, while trypsin cleaves bonds next to the basic amino acids arginine and lysine. Thus, because of the different enzyme specificity, the site of digestion will determine the type of peptides released during gastrointestinal digestion, and consequentely, their physiological properties. The physicochemical properties and primary structure of peptides of food proteins may also influence the sequence and activity of liberated peptides [16]. Moreover, Moughan and coworkers observed that peptide hydrolyzates undergo faster gastric emptying and intestinal absorption of their constituent amino acids than their whole source proteins [17]. Nguyen et al. reported a lower degree of hydrolysis of soybean proteins contained in soy-based infant formulations than that for milk protein-based infant products [18]. This could be due to the hydrophobic β-sheet structures of soy protein that made its digestion difficult. Moreover, the effect of processing on protein digestion have been studied. Drulyte and Orlien reported the effect of domestic (soaking, cooking, and baking) and industrial (autoclaving, baking, and extrusion) processing methods on the legume protein digestibility [19]. Overall, the protein digestibility increases after processing by the different methods. Since both the type of legume and the applied methods differ, it could not be concluded which specific methods were better for the individual legume type. Recently, Deng et al. have reported that the heat treatment of β-lactoglobulin provokes structural modifications on the protein that increase its gastric digestibility and transport across intestinal epithelial cells in comparison with the native protein [20]. Therefore, the chemical and structural characteristics of protein in the natural form, in processed foods, or in purified form have an undeniable impact.

The digestive procces is closely controlled by hormonal and neural regulatory mechanisms [21,22]. Digestive hormones may enhance or inhibit the secretory activity of glandular organs and the contractions of smooth muscles. Moreover, both autonomic and enteric nervous systems are involved in the regulation of digestion processes. A faster gastric emptying rate may alter the incretin and enterogastrone response, but it may also modify the hormonal response to feeding [23]. Calbet and Holst studied the influence of the degree of protein fractionation on gastric emptying, gastric secretion, amino acid absorption, and enterogastrone response, after the intragastric administration to volunteers of complete cow milk proteins or their respective peptide hydrolyzates [14]. While the rate of gastric emptying and the plasma glucagon-like peptide-1 (GLP-1) and peptide YY (PYY) responses to feeding were found to be independent of the degree of protein fractionation, amino acid composition, or protein solubility, the glucose-dependent insulinotropic polipeptide (GIP) response was accentuated when milk proteins are ingested as hydrolyzates.

Bioactive peptides acting at the local system may also affect the gastrointestinal process. Major studies have focused on animal proteins-derived peptides whose opioid agonist and antagonist activity is responsible for their modulatory effects on the gastrointestinal motility and secretion of gut regulatory signals, controlling food intake and satiety [24]. Dalziel et al. found that whey protein concentrates decreased motility in the colon, while their corresponding hydrolyzates increased the frequency of contrations [25]. Domenger and coworkers reported that hemorphins, a group of opioid peptides encrypted in the beta-chain of hemoglobin, specifically, LLW-YPWT, LW-YPWT, W-YPWT, W-YPWTQRF, and YPWTQRF had effects on intestinal peristalsis, appetite, and food intake regulation [26].

#### 2.1.2. Models to Evaluate Digestibility

The human and animal models provide the most physiologically relevant data on the digestion of protein/peptides [27]. This can be achieved by aspiration of the digestion content from the stomach [28], small intestine [29], or both [30], using imaging technologies [31] and wireless telemetric systems [29,32]. Different animals have been used for these models, including dogs, chickens, rats, and pigs [33]. Pig is the most suitable animal model to predict protein digestibility in humans because its enzymes and the physiology of its digestive tract are the closest to those in humans [34]. However, to date, few studies have evaluated the in vivo digestion behavior of bioactive peptides; milk proteins have been the most studied [35,36]. Table 1 summarizes examples of recent studies on gastrointestinal models used to evaluate the digestion of bioactive peptides derived from milk proteins.

Animal models generally involve animal death or surgical approaches in which cannulas are placed into digestive organs to access the content of the gastrointestinal tract. This associates in vivo studies with significant ethical restrictions, in addition to their high cost and long duration. In the last few years, to overcome these limitations, several in vitro gastric and small intestinal models have been developed and optimized. 

Static models are cheap, easy to use, and do not require specific equipment, thus being the most widespread used digestive systems. They consist in a series of bioreactors mimicking the physicochemical and enzymatic conditions of each digestive compartment. The most simple and common in vitro gastric digestion model is a water bath incubation model that may also include various digestive enzymes (amylases, proteases, and lipases), mucins, and salts, depending on the food product under investigation and the region of the gastrointestinal tract. Samples are commonly mixed with a simulated digestion fluid (at an appropriate pH and enzyme concentration) and incubated at 37 °C up to 2–3 h [50]. Some models employ agitation in an attempt to mimic the peristaltic movements present in the gastrointestinal tract [51], and/or a pH meter to record pH of the digest at regular intervals throughout the digestion process [18]. Numerous in vitro protocols have been described in the literature differing in the experimental conditions (pH and duration of the different steps, amount of digestive enzymes and bile salts, etc.), making the comparison of results very difficult. Recently, an international consensus of the digestion conditions was reached within the European COST Action Infogest (http://www.cost-infogest.eu/) [52,53]. This standardized method has been applied to assess the digestion of different proteins, mainly milk proteins, showing improved inter-laboratory reproducibility and consistency in comparison with in-house methods [54]. Moreover, the validation of this protocol with in vivo data has been recently performed [37,39]. However, the static models are oversimplified, are restricted to studying end digestion products, do not allow tracking the kinetic behavior of food proteins and peptides during digestion, and do not take into account the dynamic aspects of the digestive process. These limitations have prompted the development of dynamic models. Recently, Mulet-Cabero et al. reported an intermediate model built upon the harmonized INFOGEST static model and including gradual acidification, fluid and enzyme secretion, and emptying [55]. 

Dynamic systems are either mono-compartmental (simulate one compartment of the gastrointestinal tract) or multi-compartmental (several compartments). The different available systems available have been described by Guerra et al. [9], Dupont et al. [56], and Giromini et al. [57]. These models can perform continuous changes in pH and the sequential secretion of gastric or pancreatic juices, in addition to consider gastric emptying or the removal of digestion products. Therefore, because of their capacity to mimic both mechanical and enzymatic transformations occurring during gastrointestinal digestion, dynamic models are considered the best systems to reproduce the physiological conditions. 

The main mono-compartmental models are the Dynamic Gastric Model (DGM) and the Human Gastric Simulator (HGS). The DGM was developed at the Institute of Food Research (Norwich, UK) according the physiology of the human stomach [58]. It has a truncated stomach shape where the gastric emptying is regulated by a valve that allows the smallest particles to leave the stomach, whereas the bigger ones are refluxed into the top chamber to be further digested. It has been shown to accurately replicate the antral grinding forces observed in vivo [59]. This model has been applied to evaluate the digestion of human milk proteins [60], although no bioactive peptides were identified. The HGS model from the University of California, Davis (USA) provides a pattern of stomach forces comparable to the in vivo situation [61], where the emptying is achieved by batches. Its design and construction have been reported in the Master Thesis of Phinney [62]. This model has been used to study the gastric digestion of rice and apples proteins [61] and whey proteins [63]. Both mono-compartmental models are particularly relevant for gastric digestion studies; thus, the released products may be used for their subsequent digestion at the intestinal level. 

The main bi-compartmental models simulate the luminal conditions of the stomach and proximal small intestine. Based on in vivo data, these computer controlled systems reproduce the temperature, pH changes in the gastric and duodenal compartments, gastric emptying, pepsin, pancreatic juice and/or bile salts content [64,65], and dialysis of digestion end products [66]. 

Different multi-compartmental models are available. TIM-1 and TIM-2, and the Simulator of the Human Intestinal Microbial Ecosystem (SHIME^®^) were developed more than 20 years ago. TIM-1 and TIM-2 were designed at the TNO Nutrition and Food Research Center (Zeist, Netherlands) by Minekus et al. [67,68]. They are composed of the stomach and the three parts of the small intestine, integrating key parameters of human digestion including temperature, kinetics of gastric and intestinal pH, gastric and ileal deliveries, transit time, peristaltic mixing and transport, sequential addition of digestive secretions, and passive absorption of water and small molecules through a dialysis system. More than 100 papers have been published applying this digestion system for drugs, foods, and micro/macronutrients. Nowadays, this model is under continuous optimization processes, aiming at simulating infant or elderly gastrointestinal conditions [69,70,71], and developing the advanced gastric model ‘TIMagc’ [72]. The SHIME^®^ model was developed at Ghent University (Belgium) [73]. The system is constituted by five reactors simulating different parts of the gastrointestinal tract. Using the so-called M-SHIME^®^, it is possible to mimic the mucosal microbial colonization by the incorporation of mucin-covered microcosms [74]. This model was successfully used to study the effect of whey retentate on the microbial community [75]. The DIDGI^®^ system has been recently built up at the “Institut National de la Recherche Agronomique” (INRA, France). It consists of two consecutive compartments simulating the stomach and the small intestine. It is equipped with temperature, pH and redox sensors, and variable speed pumps to control the flow of meal, HCl, NaHCO_3_, bile, and enzymes, as well as the emptying of each compartment. Flow rates are regulated by specific computer-controlled peristaltic pumps. Anaerobic conditions can be simulated by purging air with nitrogen, and a Teflon membrane with 2 mm holes is placed before the transfer pump between the gastric and the intestinal compartment to simulate the sieving effect of the pylorus in human [76]. This model has been validated and applied for the digestion of proteins from infant formulas [77], bovine skim milk [41], and human milk [78,79,80]. The SIMGI^®^ was developed in The Institute of Food Science Research (CIAL, CSIC-UAM) at Madrid, Spain. The stomach compartment is constituted by a methacrylate jacket with a flexible silicone membrane, where pressure is applied by water in peristaltic cycles. Recently, this model has been used to study the gastric digestion of a whey protein concentrate, comparing the results with those found with the INFOGEST static protocol [81]. These authors demonstrated that the protein digestibility and the peptide profile depended on the sequential addition of pepsin, the peristaltic movements, and the gastric emptying. 

All these models are relatively complex, and their set up and maintenance costs are very high. Moreover, they require the programming of the major parameters based on in vivo data to accurately simulate in vivo digestion conditions [56,77]. Ultimately, in vitro models must be compared to in vivo models to establish their accuracy [33]. Recent advances in non-invasive medical technologies and computational fluid dynamic tools have opened new opportunities to better understand the physicochemical conditions occurring during food digestion [82]. Complete in vivo processes, particularly hormonal and nervous control, feedback mechanisms, mucosal cell activity, peristaltic movements, and immune characteristics need to be mimicked [9]. Therefore, a combinatorial approach involving in vitro models and human cultured intestinal cells to simulate both digestion and absorption processes has been proposed.

### 2.2. Absorption of Food Peptides

Absorption of most of the digestion products occurrs in the jejunum, where chyme enters from the stomach, and it is further broken down into nutrients (including peptides, fatty acids, mono- and oligosaccharides, vitamins, and minerals) that cross the intestinal wall, reaching the systemic circulation. Traditionally, it was thought that once ingested, all peptides and proteins were hydrolyzed by digestive enzymes to their constituent amino acids that were absorbed across the intestinal epithelial barrier. It was also believed that proteins and peptides were only absorbed under pathological conditions. However, in the last few years, it has been found that many peptides are absorbed by intestinal cells under normal conditions, being detected in both newborn and adults’ bloodstream and/or target organs where they exert their biological activities [83,84,85]. 

To date, four different routes of peptides absorption have been described: paracellular diffusion, transcellular passive diffusion, transcytosis, and carrier-mediated transport. Following, the main characteristics of these absorption pathways and examples of peptides using them are summarized. 

(a) Paracellular diffusion involves the movement of molecules via water-filled pores/channels between cells. Approximately 0.01–0.1% of the total intestinal surface area consists of water-filled pores that corresponds to 200–2000 cm^2^. This surface is large enough for the absorption of small quantities of peptides (pM–nM range); thus, it is adequate to exert their biological activity [86]. The paracellular pathway is regulated by tight junctions (TJs) that separate the apical and basolateral membranes of the epithelial cells. TJs are multiprotein complexes containing zonula occludens-1, occludin, and claudin proteins that form a firm biological barrier restriciting the paracellular flux of water, ions, and solutes [87]. TJs prevent the crossing of substances through the space between plasma membranes of adjacent cells, and they restrict the process of polar macromolecule penetration [88]. Therefore, peptides passing through the intestinal epithelium enter the cells by diffusion or active transport. This transport is mainly dependent on the physicochemical properties of the peptide such as molecular weight and ionic charge [86]. Thus, this route has been reported as that preferred by hydrophilic negative charged low-molecular-weight peptides [89,90,91]. A variety of bioactive food oligopeptides have been found to be transported by passive diffusion via paracellular TJs (see the review of Xu et al. [92]). Moreover, this route was used by the 43-amino-acids peptide lunasin and its fragment RKQLQGVN, which is released during lunasin’s gastrointestinal digestion, to cross intestinal epithelial barrier [93].

(b) Transcellular passive diffusion involves the transport of molecules through apical and basolateral membranes in a concentration-based and energy-independent manner [94]. The transport of bioactive peptides through passive diffusion is dependent on peptide characteristics such as size, charge, and hydrophobicity [95]. Thus, because of the composition of the cell membrane by a lipid bilayer, it is widely accepted that lipophilicity plays a key role in this transport mechanism. While hydrophilic peptides prefer paracellular diffusion to cross the intestinal epithelium, transcellular transport is the chosen route by lipophilic peptides. Other factors, such as the peptidic chain length and number of polar groups also seem to determine the passive diffusion of bioactive peptides. Moreover, the transcellular absorption of a peptide depends on the energy required to break water–peptide hydrogen bonds, allowing the molecules to enter the cell membrane [96].

(c) Transcytosis involves the energy-dependent transport of material from one side of the polarized cell to the other. This route includes apical endocytotic uptake, transcytotic transport via internalized vesicles called endosomes, and basolateral secretion [97,98]. Since peptides need to interact with the apical lipid bilayer of epithelial cells through hydrophobic interactions before being internalized by the cells, transcytosis seems to favor the transport of long-chain (more than four amino acid residues) and hydrophobic peptides [99,100]. Thus, a recent study has suggested that the high content of hydrophobic amino acids in the antioxidative peptide YWDHNNPQIR could determine its transport across Caco-2 cell monolayers via transcytosis [101]. In addition to the importance of hydrophobicity, other factors have also been recognized as determinants in the transcytosis transport of peptides. Thus, cell models have reported that the number of polar groups and the net charge of peptides, especially the positive charge, show positive effects on their transcytosis transport [102,103].

(d) Carrier-mediated transport involves the movement of peptides against the concentration gradient, which is mediated by specific cell membrane proteins that function via anti-, sym-, and uniporter mechanisms. Antiporters translocate peptides in opposite directions, whereas symporters transport them via cotransport in the same direction over the blood membrane. Uniporters function unidirectionally, without cotransport [94]. This transport system is dependent of susbtance concentration, susceptible to inhibition, and specific to the molecules’ structure [104]. Among peptide carriers, transporter 1 (PepT1) is a high-capacity and low-affinity carrier that drives peptides from the gastrointestinal lumen into the intestinal epithelium in a proton gradient and membrane potential-dependent manner [105,106,107,108]. Although it has been described that PepT1 preferentially binds short-chain bioactive peptides, specially di- and tri-peptides, with neutral charge, and high hydrophobicity, it has also been found to be able to recognize dipeptides with an extreme bulk or two positive charges [109]. However, PepT1 is unlikely to bind hydrophilic or hydrogen regions [110]. Recently, Wang and Li used a Caco-2 cell monolayer model to study the transport routes chosen by casein-derived peptides [89]. These authors found that PepT1 was responsible for transporting low molecular weight peptides, while high molecular weight casein peptides crossed the intestinal barrier through paracellular diffusion. Moreover, the bioavailability of peptides transported by PepT1 was higher than those transported through the paracellular route. PepT1 has also been described as the carrier of angiotensin converting enzyme (ACE) inhibitory peptides IPP and LKP, which were released from milk β-casein and fish/chicken muscle protein, respectively [111], as well as of other food bioactive peptides [101,112,113,114,115,116]. In addition to PepT1, other peptide carriers present in the basolateral membrane have been suggested to participate in the transport of hydrolysis-resistant small peptides into blood. However, this fact has not been proven yet [117,118]. 

#### 2.2.1. Limitating Factors for Peptides Absorption

In addition to the peptide characteristics such as length, primary and secondary structures, hydrophobicity/lipophilicity, and charge that influence on the process of peptide transport across the intestinal wall, other factors have been reported to limit/promote the absorption and bioavailability of food-derived peptides [119,120]. Firstly, food processing can provoke undesiderable reactions between peptides and co-existing compounds present in the food matrix, limiting peptides’ absorption [121,122]. However, the available mechanistic data are still very scarce. In a placebo-controlled crossover human study, Foltz and coworkers reported a delay in the bioaccessibility and plasma clearance of antihypertensive peptide IPP present in yogurt when it was consumed as the base of the breakfast meal in comparison with the delivered peptide in a fasted state [83]. Recently, Lacroix et al. [123] have reported the higher degradation of dipeptidyl peptidase IV (DPP-IV) inhibitory peptides from whey proteins by peptidases present at the apical side of Caco-2 cell monolayers when incorporated in a matrix containing inorganic salts and glucose. Furthermore, interactions between free radicals formed from food phenolic compounds through oxidation reactions and nucleophilic moieties of peptides can result in new peptide derivatives, thus modifying their bioavailability [121,122]. In addition, the absorption of peptides can be modified as result of the effects of co-existing compounds in the food matrix on the peptides transport route. Thus, compounds that use the same PepT1 transport pathway as small peptides can compete with them, reducing their absorption rate [124]. Antioxidant black tea polyphenols have been demonstrated to down-regulate PepT1 expression, resulting in a decrease in the dipeptides absorption across Caco-2 cell monolayers [125], while this transport was favored by dietary amino acids and protein hydrolyzates that up-regulate PepT1 expression [126]. 

The peptide digestibility and bioavailability can also be modified by alterations in the luminal environment, intestinal barrier function, and gut microbioma provoked by food components [127,128]. Even peptide products resulting from the degradation of dietary peptides by digestive enzymes can act locally, potentiating the action of other bioactive peptides by improving pathway signaling, as well as the morphology and functionality of intestinal cells [128,129]. Detailed peptidomics and transcriptomics studies focusing on investigating the bioaccesibility and bioavailability of bioactive peptides into the complex food matrix and the development of enhancement strategies of peptides stability would eliminate the need for the intense purification of protein hydrolyzates. In addition to food properties, other non-dietary factors also affect the bioavailability of bioactive peptides. It has been demonstrated that an altered intestinal environment provoked by diseases such as ulcerative colitis or colon cancer, pharmacological treatments, or endogenous hormones can influence the peptides’ absorption [130]. Thus, these physiological factors should be taken in account to interpret data from in vivo studies focused on assessing the bioavailability of bioactive peptides.

In addition to the digestive enzymes present in the gut lumen and the brush border peptidases of the microvilli that can degrade bioactive peptides into smaller molecules and free amino acids, other barriers are encountered by peptides during their transit through the gastrointestinal tract that can affect their absortion rate and bioavailability. The first physical obstacle found by peptides is the mucus barrier, which is a hydrogel layer composed of large glycoproteins, mainly mucins, whose major function is to lubricate the passage of chyme and to protect the epithelium against mechanical damage and pathogens [131,132]. The internal surface of the small intestine is covered by a monolayer of epithelial cells, mostly enterocytes, and mucus-secreting goblet cells. The apical side of enterocytes, called the brush border, is covered by microvilli that increase the intestinal surface area involved in the digestion and absorption of molecules [133]. Once peptides reach the epithelium, they can be degraded, transformed, or absorbed, depending on their properties. Below the intestinal epithelium, peptides find the subepithelial tissue divided into the lamina propria and mucosa muscularis. In vivo, this highly vascularized connective tissue is not a limiting barrier to the absorption of small peptides [134]. 

#### 2.2.2. Models of Peptide Absorption

To measure the in vitro bioavailability of peptides, two major approaches are currently used. The common strategy without using cells involves the simulation of gastrointestinal digestion of proteins/peptides with digestive proteases (as explained in Section 2.1.) combined with the use of semipermeable membranes of different cut-off values (1–10 kDa) during the intestinal phase to estimate the content of peptides available for absorption [135]. However, because of the lack of reproducibility of these cell-free systems, in the last few years, new approaches combining in vitro gastrointestinal digestion and intestinal cell models have been optimized and applied for estimation of the bioavailability of peptides. The American Type Culture Collection (ATCC) offers a broad array of commercially available human intestinal cell lines, of which Caco-2 and HT-29 cells are the most commonly used [94].

Caco-2 cells are derived from human colonic adenocarcinoma, but once cultured on semipermeable inserts, they differentiate into an enterocyte-like phenotype, with a characteristic apical brush border with microvilli, TJs, digestive proteases, and active receptors and transport systems [119,136]. Since its introduction in the 1990s [137,138,139], the use of this cell model for permeability studies in drug discovery and development as well as in the drug absorption, distribution, metabolism, and excretion (ADME) sciences has exponentially risen. In the field of bioactive peptides, over the past 10 years, a high number of studies have evaluated the absorption of these peptides and the mechanisms of transport across Caco-2 cell monolayers (Table 2). Thus, the absorption of intact sequences across Caco-2 cells monolayers has been demonstrated for antioxidant peptides derived from soybean protein [140], corn gluten [141], milk proteins [89,142,143], and dry-cured Xuanwei ham [144], and for antihypertensive peptides derived from lactoferrin [145], ovotransferrin [114], and ovoalbumin [146]. Similarly, this model has been used to demonstrate the efficient transport of multifunctional soybean peptide lunasin or its derived fragment RKQLQGVN by paracellular diffusion [96], and of multifunctional peptides released from lupin storage proteins by digestive enzymes [147]. However, in spite of the numerous articles published, the conditions used such as cell density, seeding time, or time of incubation with the samples differ among them, making the comparison of the results difficult. Therefore, inter-laboratory studies should be needed to harmonize and improve the overall data interpretation. Moreover, although differentiated Caco-2 cells present a proteome expression similar to jejunal enterocytes, important differences exist between both cells. Thus, it has been demonstrated that the level expression of brush border enzymes is lower in Caco-2 cells, being some of these proteinases and peptidases below the limit of detection. This makes the metabolism of peptides lower when they are cultured with Caco-2 cells compared to that in the human intestine [88].

Caco-2 cells are also characterized by the absence of a protective mucus layer that, sometimes, is compensated by the co-culture of these cells with different mucus-secreting globet cells. The sub-culture Caco-2/HT-29 has been recognized as a more physiological relevant model compared to Caco-2 monoculture because Caco-2 cells provide the barrier function and absorptive enterocyte population, and HT-29 cells present the mucus producing goblet cells [168]. This co-culture model has been employed to evaluate the absorption of peptides from whey proteins [158] (Table 2). However, these two cell lines do not mix well in co-culture, and HT-29 tend to grow as colonies embedded in Caco-2 cells, resulting in an irregular mucus layer [169]. This makes Caco-2/HT-29 co-cultures showing lower transepithelial electrical resistance (TEER) and increased permeability compared with Caco-2 mono-cultures [169,170]. Moreover, HT-29 mono-cultures are not suitable for absorption studies due to their inability to form a tight barrier when cultured under standard conditions. However, prolonged cultivation under modified culture conditions leads to the generation of subclones such as HT-29-MTX, which is produced after the addition of methotrexate to the culture media [171]. This subclone is able to form a tight monolayer and express brush border enzymes, becoming a common model to study the role of goblet cells on peptide absorption [162]. Another aspect limiting the utility of Caco-2 cells for studies of peptide absorption is the low level of endocytosis and transcytosis due to the reduced level of expression of caveolins, which are proteins that are involved in these mechanisms of transport [88].

This limitation has been solved by co-culturing Caco-2 cells with human hematopoietic Raji B cells that induce an M-cell-like phenotype in Caco-2 cells, stimulating the expression of caveolins and the transcytosis capacity of the intestinal cells [172,173]. In recent years, three-dimensional culturing systems are being developed combining physiological relevant parameters such as microbiota and shear stress with peristaltic intestinal movements. In these systems, called gut-on-a-chip, Caco-2 cells are cultured on a semipermeable membrane with the apical and basolateral sides facing two different fluid-filled channels simulating intestinal lumen and blood, respectively. Moreover, important changes in the cell phenotype such as mucin expression and the formation of villous- and crypt-like structures are induced by pressure pulses to mimick both heart beat and peristaltic movements [174,175]. Although these systems are generally accepted for their ease of use and moderate cost, their application for peptide absorption studies is still limited, and further studies confirming their utility are needed. On the contrary, three-dimensional intestinal mini-guts, also called organoids, are presented as a promising in vitro model for nutrient and drugs absorption, enteroendocrine secretions, and intracellular signaling [176]. Organoids, as obtained from isolated intestinal tissue, contain all types of intestinal epithelium cells and exhibit most of the epithelium functional properties, including absorptive functions. The potential of this model to reduce the number of animal experiments and complement the existing information of peptide absorption has been suggested [177]. Thus, organoids have been successfully applied to study the absorption and transport of dipeptides [178]. However, no studies on the application of this model for studying the bioavailability of food bioactive peptides are yet available.

It has been recognized that the cell-based experiments used to determine the kinetics and systemic activities of bioactive food peptides are not consistent with in vivo data because the doses used are generally higher than those used in animal and human models, and the in vitro studies do not consider digestive and metabolic processes that occur before peptides reach their target organs [179]. Thus, in the last years, the necessity of in vivo models considering the ADMEs features of bioactive peptides at physiologically relevant concentrations and times has been emphasized. However, although several animal and human studies have provided evidence on the absorption of bioactive peptides (see the recent review of Xu et al. [92]), the existing data on their stability, kinetics, and bioavailability are still scarce. To design rationale and consistent animal and human studies, different aspects need to be taken into account. Firstly, potential interactions between bioactive peptides and other components of the food matrix may result in changes in the bioavailability and bioactivity of food peptides [2,179]. Moreover, safety issues should be considered to avoid toxic effects at the doses used [179]. The inter-individual variability due to age, sex, race, and/or diseases may also influence the bioavailability of bioactive peptides [180,181]. Finally, the development of optimized techniques to identify and quantify bioactive peptides in plasma and organs at low concentrations will be required to increase the sensibility of human studies.

### 2.3. Effects of Gastrointestinal Endogenous Protein-Derived Peptides

Gut endogenous proteins represent a larger and more constant supply of protein in the gastrointestinal tract in comparison with dietary protein [182]. They are constituted by gastrointestinal tract epithelial turnover, gut microflora proteins, and soluble secreted proteins such as mucins, digestive enzymes, hormones, serum albumin, immunoglobulins, and lysozymes, among others [183]. Although these endogenous proteins have been exhaustively studied to estimate the dietary amino acid requirements and digestibility, the data on their potential as a source of bioactive peptides are still scarce. However, given the high amount of endogenous proteins present in the gastrointestinal tract, a wide array of potentially bioactive peptides are expected to be liberated during the digestion process. In a preliminary in silico gastrointestinal digestion prediction model, 26 gut endogenous proteins were evaluated as a source of bioactive peptides [184]. The total number of bioactive peptides predicted to be released ranged from 1 (secretin) to 39 (mucin-5AC), of which ACE-inhibitory peptides were the most frequently observed. These results were confirmed by an in vitro digestion assay of endogenous proteins, resulting in the release of a high number of antioxidant, ACE, and DPP-IV inhibitory peptides [185,186]. Similarly to dietary bioactive peptides that have been demonstrated to bind to specific receptors in the gut modulating gut motility, satiety, and the secretion of gastrointestinal endogenous proteins, peptides released from these endogenous proteins might also have effects on gut physiology and functions [187]. Therefore, little modifications of both dietary and endogenous sources of bioactive peptides offers a great opportunity to modulate gut processes.

### 2.4. Strategies to Improve Bioavailability of Food Peptides

Once the mechanisms involved in the transport of bioactive peptides and the factors influencing their absorption are known and understood, it is possible to design valuable strategies that improve the bioavailability of peptides and maintain their potent in vivo bioactivities. These strategies aim at achieving the following objectives: (i) reduction of the detrimental effects of food processing on peptides bioactivities; (ii) promotion of the desiderable interactions between peptides and other food matrix components, reducing the undesiderable ones; (iii) protection of bioactive peptides from gastrointestinal conditions and digestive enzyme activity; (iv) control of the sustained peptides’ release directed at their target organs; and (v) improvement of the transport of bioactive peptides across the intestinal epithelium and target cells [188].

Following, the existing evidence on systems developed and currently applied to enhance the absorption and bioavailability of food-derived bioactive peptides is summarized.

#### 2.4.1. New Food Processing Techniques

Thermal processing techniques, including sterilization, pasteurization, drying, and evaporation, have been generally used to ensure food preservation and microbial safety. However, the detrimental effects that the temperature provokes on food components, specifically damaging the structure of peptides and affecting their bioactivity, are well known. Thus, in the last few years, novel non-thermal techniques have been designed and optimized to mitigate these negative effects and enhance the bioaccesibility, bioavailability, and bioactivity of food-derived peptides [122]. Strategies such as ultrahigh hydrostatic pressure, pulsed electric field, microwave, irradiation, and ultrasound have the ability to inactivate microorganisms at near-room temperature, preserving the sensory, functional, and nutritional quality of food products [189]. However, due of the current limited data on the potential effects of non-thermal processing techniques on the bioavailability and bioactivity of food peptides [190], further studies should be conducted. 

#### 2.4.2. Modifications in Peptides Structure and Properties

In order to protect food-derived peptides from the action of digestive enzymes, improve their intestinal permeability, or retain/potentiate their biological activity, different modifications of the peptide’s structure have been suggested. Changes in N- and C-amino acid terminals by acetylation/amidation have been demonstrated to protect the peptide from the action of amino- and carboxipeptidases, respectively [191,192]. Cyclization is another particular biochemical feature that increase peptide resistance to exopeptidases degradation, enhancing their stability and half-life in the gastrointestinal environment [193]. In addition, modifications inside the peptide chain can result in improvements of the biological activity of peptides. Thus, the phosphorylation of hydroxyl groups of serine in caseinophosphopeptides prevent their hydrolysis by digestive enzymes, enhance their absorption, and protect their mineral-binding capacity [194]. Tanzadehpanah et al. investigated the effect of two peptides (KDEDTEEVP and KDEDTEEVH) differing in the last amino acid [195]. The presence of proline favored the ACE-inhibitory activity of peptide KDEDTEEVP, while the highest antimicrobial and antioxidant activities were shown by the peptide KDEDTEEVH. Changes in the structure and molecular mass of peptides-based drugs have also been suggested to have a positive impact on their permeation across the intestinal mucosa [196,197]. However, data about the influence of these modifications on the absorption capacity of food derived peptides are not still available. 

#### 2.4.3. Protease and Peptidase Inhibitors

The co-administration of peptides with protease/peptidase inhibitors can prevent peptides from degradation in the gastrointestinal tract, thereby facilitating intestinal absorption [198,199,200]. Although synthetic enzyme inhibitors have been widely used to protect peptide-based drugs, naturally occurring inhibitors such as Bowman–Birk protease inhibitor (BBI) and Kunitz trypsin inhibitor are currently preferred because of their lower side effects and compatibility with food derived peptides. Thus, BBI has been demonstrated to protect soybean peptide lunasin from in vitro gastrointestinal digestion, improving its absorption capacity and bioavailability [201]. However, major drawbacks have been associated with the use of protease inhibitors. As a result of their own susceptibility to enzymatic degradation in the gut, high doses need to be co-administered with peptides to exert protective effects. The chronic and prolonged use of high doses of enzyme inhibitors may alter the metabolic pattern of the gastrointestinal tract, leading to the inappropiate digestion of nutritive proteins, and they can also provoke an endogenous regulatory mechanism stimulating the production of digestive peptidases [96].

#### 2.4.4. Absorption Enhancers

Absorption enhancers are substances that allow bioactive compounds to permeate across the intestinal epithelium into systemic circulation and reach the target organ to exert their biological activity [202]. Several mechanisms of action have been described for absorption enhancers, including the short-term disruption of the structural integrity of the intestinal barrier, the reduction of the mucus viscosity, the aperture of TJs, and the increase of the membrane fluidity [96]. They should be safe, pharmacologically and chemically inert, non-toxic, non-irritant, and non-allergenic [203,204]. However, similarly to protease inhibitors, the major limitation of the long-term use of penetration enhancers is the potential damage to intestinal membranes, resulting in local inflammation [205]. Moreover, the administration of these permeation enhancers can potentially introduce undesiderable substances into the bloodstream [188].

Among absorption enhancers that are potentially useful to improve the oral bioavailability of proteins and peptides, the most commonly investigated are surfactants, chelating agents, bile salts, cationic and anionic polymers, and fatty acids and their derivatives. Although permeation enhancers have been widely used in the pharmacological field, improving the bioavailability of peptide-based drugs such as insulin, interferon-gamma, and recombinant human growth hormone [206], their application in the food industry is still very limited. However, some food-grade permeation enhancers, such as citric acid, fatty acids, and chitosan are recognized promising candidates to be applied in the development of bioactive peptides-based functional foods and nutraceuticals. 

#### 2.4.5. Delivery Systems of Food Bioactive Peptides

As it has been previously described in this review, food bioactive peptides are labile compounds that are sensitive to the light, oxygen, and temperature, capable of interacting with other components when they are dispersed in the food matrix, and susceptible to the harsh conditions of the gastrointestinal tract. Therefore, similarly to other nutrients and food bioactives, the incorporation of peptides into carriers has been demonstrated to increase their stability and bioavailability [207]. These systems show multiple mechanisms of action such as mucoadhesion, permeation enhancement across absorptive epithelium and TJs, high total surface area, targeted delivery, and affinity to specific intestinal cells [208]. Effective delivery systems must protect bioactive peptides from adverse conditions (variations of pH and temperature, enzymatic activity, etc.) inherent to food processing, storage, and gastrointestinal digestion. Moreover, these systems must be able to maintain peptides’ activity and stability until their controlled and sustained release at the target site. Additionally, they should not modify the physicochemical and organoleptic properties of the peptide-based food products. Finally, because of the high demand by consumers of “clean-label” food products, carriers manufactures are limited to the use of natural ingredients and biodegradable, generally recognized as safe (GRAS) alternatives [209,210]. This has made it much more difficult to produce food-based delivery systems with the required functional attributes. Therefore, the design of the most suitable delivery systems requires extensive knowledge of the physicochemical and molecular attributes of bioactive peptides (molecular dimmension, electrostatic effects, polarity, solubility, and surface activity) as well as the environmental factors, ingredients interactions, and digestive conditions that can negatively alter the structure and bioactivity of peptides [210,211]. The most recent delivery systems applied for the encapsulation of food bioactive proteins and/or peptides and the effects on their stability, bioavailability, and bioactivity are summarized in Table 3.

Among the factors determining the encapsulation efficiency, the peptide charge, the type and purity of the carrier material, and the core-to-wall ratio have been extensively investigated [212]. The encapsulation of negatively charged intact proteins such as bovine seroalbumin has been reported to result in lower encapsulation efficiency (34%) than that found for liposomes prepared with positively charged lactoferrin (46%) [213]. Low encapsulation efficiency has been also described for negatively charged caseinophosphopeptides [212]. The kind and purity of carrier material are also recognized important factors that determine encapsulation efficiency, although information on the encapsulation of protein hydrolyzates or peptides is very limited, making it difficult to draw final conclusions. Lipids, polysaccharides, and protein-based carriers have been employed for the encapsulation of food bioactive peptides. Among lipid-based systems, lipospheres are appropriate for encapsulating hydrophobic peptides, while liposomes are compatible with a wide variety of bioactive peptides of hydrophilic, hydrophobic, and amphiphilic nature [214]. This breadth of utility along with the lesser content of high saturated fatty acid content has made liposomes gain popularity as a delivery system of bioactive peptides (Table 3). However, several shortcomings have been associated with the use of liposomes in the functional food industry such as their thermal instability, which limits their use in processed food products, the cholesterol added to increase the stability of the system, and the risk of lipid oxidation during the production, processing, and storage of the food products [215]. Therefore, in order to develop the most suitable lipid-based systems to encapsulate bioactive peptides, both health and product quality challenges must be considered and overcome.

The structural stability, abundance in nature, and low cost have made polysaccharides ideal delivery agents for food bioactive compounds. Polysaccharides derived from animals, plants, and microorganisms, such as Arabic gum, chitosan, cyclodextrin, and maltodextrin have been commonly used for food proteins and peptides encapsulation (Table 3). Chitosan is a natural non-toxic, biocompatible, and biodegradable polymer [248]. As a result of its permeation-enhancing effects, chitosan-based systems have been recognized as among the most promising matrices for bioactive peptide entrapment and delivery. Thus, these systems have been recently proven to increase the in vivo antihypertensive efficacy of stone fish protein hydrolyzates [229,230] and to retain the antioxidant activity of rainbow trout protein [232] and seed protein [245] hydrolyzates. Additionally, tripolyphosphate (TPP) is the most accepted crosslinker to prepare chitosan hydrogels through ionic crosslinking [249,250]. Consequently, combinations of chitosan with TPP have attracted the interest because of their properties such as biocompatibility and mucoadhesiveness, and their soft preparation procedure [251]. Thus, chitosan–TPP combinations under optimized conditions have been described as good peptides entrapment systems with controlled properties [237]. Maltodextrin is other commonly used polysaccharide-based carrier, alone or in combination with Arabic gum to retain the biological activity of casein [252], flaxseed protein [239,241], and *Phaseolus lunatus* protein [242] hydrolyzates.

Despite the popularity of protein-based carriers for delivering other food bioactive compounds such as flavonoids, vitamins, and β-carotene, their use in bioactive peptide encapsulation is limited. The main reason is the instability caused by the structural similarity of core and wall materials [252]. Soybean and whey protein isolates have become the main alternative to encapsulate bioactive peptides from casein and whey proteins [253,254], whereas actylated and high-pressure treated rapesed protein isolate has been used to encapsulate peptides from the same vegetal material [255].

The purification of carrier materials provides an additional important advantage to increase the encapsulation efficiency. However, this does not seem to be economically possible in the functional food industry because of the extra cost of obtaining and purifying processes of the wall material [256]. Thus, further studies are needed to identify and adapt processes increasing the encapsulation efficiency for bioactive peptides without increasing the costs. Generally, encapsulation involves the use of higher amounts of wall materials than the active core compounds. The core-to-wall ratio influences the encapsulation efficiency; thus, it decreases when the core concentration increases [257]. However, controversial results from studies encapsulating different protein hydrolyzates/peptides indicate that in addition to the influence exerted by the core-to-wall ratio, the nature and molecular composition of the encapsulated material, and the type of the wall material, another factor to take into account is the encapsulation technique [208]. The most relevant techniques applied for the encapsulation of food protein hydrolyzates and peptides include film hydration, spray drying, coacervation, and ionotropic gelation (Table 3). 

Film hydration is a simple and effective encapsulation procedure where phospholipids self-assemble by heating, agitation, and sonication, thereby trapping the aqueous core containing bioactive peptides. This procedure has been recently used to retain the stability, bioavailability, and bioactivity of peptide fractions from fish [234,235] and vegetal proteins [240,241] under simulated gastrointestinal conditions. In spite of the popularity of this technique, the major limitation is the uncontrolled assembly mechanism resulting in low and variable reproducibility and encapsulation efficiency. Moreover, organic solvents require to be removed prior to the use of encapsulated products in functional foods, also affecting the efficiency and increasing the costs of the process. 

Spray drying is a procedure involving the formation of droplets and spraying at high temperature that results in dried particles. This technique is commonly used to achieve encapsulation by using protein and polysaccharide-based carriers because of its low cost and simplicity. Although the biopeptides microencapsulation using spray-drying is the oldest technique used to protect them against deterioration [258], in the last few years, this procedure has applied to encapsulate bioactive peptides released from vegetal proteins by enzymatic hydrolysis [236,238]. 

Coacervation and ionotropic gelation are also efficient delivery systems based on electrostatic interactions of the encapsulation materials. As shown in Table 3, the nanoencapsulation of peptides from fish protein by ionotropic gelation has been recently reported to increase the bioavailability of encapsulated ACE-inhibitory peptides generated from stone fish proteins. Indeed, after being administrated to spontaneoulsy hypertensive rats, higher antihypertensive activity was determined for encapsulated peptides in comparison with non-encapsulated peptides [229,230]. 

In the last few years, different delivery systems (nanoliposomes from soybean lecithin, chitosan nanoparticles, microspheres, and microgels generated from alginate and chitosan) have been designed and developed, becoming promising bioactive peptides carrier candidates [211,226]. To select the most appropriate systems to encapsulate bioactive peptides, aspects such as cost, ease of fabrication, effects on product quality, stability, bioavailability and bioactivity, and organoleptic attributes should be considered. Moreover, further research on the in vivo physiological repercusion of these delivery systems is needed. 

## 3. Conclusions

It has been recognized worldwide that food-derived bioactive peptides are valuable ingredients of functional foods and/or nutraceuticals to promote health and reduce the risk of chronic diseases. However, although oral administration is the preferred route for bioactive peptides, the translation of in vitro activity to in vivo effects when peptides are orally ingested is not always realistic. These discrepancies are due to the molecular characteristics of peptides as well as to both dietary and non-dietary factors. Molecular mass, amino acid sequence, and additional structure modifications are determinant properties for the resistance of peptides to digestive enzymes and the preferred transport route to cross the intestinal barrier. Among dietary factors, the interactions between peptides and other compounds of the food matrix are considered relevant, since these components may reversibly or irreversibly react with bioactive peptides, modulating their digestibility and/or altering the routes of absorption of peptides, influencing their bioavailability. However, to date, the existing evidence on the effects of the food matrix is still limited. In addition, the behavior of peptides during their transit through the gastrointestinal tract depends on health and pathological conditions that can alter the digestive and absorptive gut environment. Thus, for a better understanding of the in vivo physiological effects of food bioactive peptides, extensive research studies on their gastrointestinal stability and transport are needed. Combined in vitro studies simulating gastrointestinal digestion conditions and cell culture mimicking the intestinal absorptive environment are being optimized, becoming an interesting and valuable approach to confirm the beneficial role of peptides on health at doses that are physiologically relevant. Due to the low bioavailability of most food peptides, efforts are being focused on the design of new strategies that increase their resistance to the action of digestive enzymes during their transit through the gastrointestinal tract and allow the controlled release of intact and active peptides in the target organs where they exert their biological activity. Among these strategies, delivery systems with natural, safe, and biocompatible materials are becoming the most promising; thus, further research should be needed to optimize the encapsulation conditions enhancing the digestibility and bioavailability of food bioactive peptides.

## Figures and Tables

**Table 1:** Some examples of recent studies on gastrointestinal models used to evaluate the digestion of bioactive peptides derived from milk proteins.

Dairy Food Product	Gastrointestinal Model	Site/Type	Outcomes	Reference
Casein and whey proteins	In vivo	Human jejunum	Identified 415 and 230 peptides from casein and whey proteins Identified β-casomorphin-7, f(60–66) and various peptides containing the same sequence, antihypertensive peptides [β-casein f(134–138) and α_s1_-casein f(143–146) and f(60–66)], hypocholesteromic β-lactoglobulin peptide f(71–75) and DPP-IV inhibitory β-lactoglobulin peptide f(9–14)	[37]
Human milk	In vivo	Infant stomach	Identified 649 β-casein-derived peptides, most of them with biological activity	[38]
Casein and whey proteins	In vivo	Human jejunum	Identified 356 and 146 peptides from casein and whey proteins Identified opioid β-casomorphins, f(57-, 58-, 59-, and 60–66) and antihypertensive peptide β-casein f(198–113)	[29]
Skim mik	In vivo	Mini-pig duodenum	Identified a high number of resistant bioactive peptide sequences	[39]
Skim milk	In vivo	Mini-pig duodenum	Identified 400 bioactive peptides with antihypertensive, anti-stress, antimicrobial, antioxidative, opioid agonist, immunomodulating anti-thrombotic, protease/peptidase-inhibitory and/or mineral-binding properties The position of cleavage sites is highly conserved, independently of the matrix ingested	[40]
Infant formula	In vivo	Piglet jejunum and ileum	Identified β-casein peptides f(60–66) and f(80–89) with immunomodulatory and antihypertensive activities, respectively	[8]
Unheated and heat skim milk powder	In vitro	Dynamic: DIDGI^®^	Identified antihypertensive α_s1_-casein peptides [f(143–149) and f(90–94)], opioid [α_s1_-casein f(90–95) and k-casein f(33–38)], and antibacterial α_s2_-casein peptides [f(183–207) and f(186–206)]	[41]
Skim milk with β-casein variants A1, A2, F and I	In vitro	Static INFOGEST with human gastric and duodenal juices	Quantitative differences in β-casomorphin 7, β-casein f(60–66) due to the different milk matrices	[42]
Skim milk with β-casein variants A1, A2 and I	In vitro	Static INFOGEST with human gastric and duodenal juices	Identified antihypertensive β-casein f(133–138), ACE-inhibitory peptides f(6–14), f(59–68), f(60–68), f(193–202), opioid peptide f(60–66) and antimicrobial and immunomodulatory peptide f(193–209)	[43]
Grana Padano cheese	In vitro	Static with porcine pepsin and porcine pancreatin (Pepn) and INFOGEST	Double number of CPPs in cheese digests using the PePn protocol in comparison with the INFOGEST method, independently of the cheese aging	[44]
Cheddar, Gorgonzola, Maasdam and Grana Padano cheeses	In vitro	Static INFOGEST	β-casomorphin 4, f(60–63) and β-casomorphin 7, f(60–66), were released from all studied cheeses	[45]
Human milk and infant formula	In vitro	Static	Similarities and differences in the post-digestion profiles of human milk and infant formula Conserved function between bovine and human milks	[46]
Commercial dairy products	In vitro	Static	Comparison between peptides with satiety-influencing and DPP-IV inhibitory properties from different dairy products	[47]
Spanish blue cheese (Valdeon)	In vitro	Static	High number of bioactive peptides, including antihypertensive, antioxidant, intestinal mucin-secretory, and antibacterial	[48]
Gamalost and Norvegia cheeses	In vitro	Static but with human gastric and duodenal juices	Both cheeses showed an increased ACE-inhibitory activity during gastric digestion. Norvegia cheese showed pronounced increased activity after duodenal digestion	[49]

ACE: Angiotensin converting enzyme; CPP: Caseinophosphopeptides; DPP-IV: Dipeptidyl peptidase IV.

**Table 2:** Recent studies on cell models used to evaluate the absorption of food bioactive peptides.

Protein/Peptide Substrate	Biological Activity	Cell Model	Absorption Study Conditions				Outcomes	Reference
			Density (cells/cm^2^)	Seeding Time (days)	Sample Concentr.	Time (min)		
Simulated digest from Alcalase^®^ soybean protein hydrolyzate	Antioxidant	Caco-2	1.2 × 10^5^	18–21	1.0 ^c^	120	Absorption of antioxidant peptides across cell monolayer	[140]
Soybean peptides IAVPTGVA, LPYP, and IAVPGEVA	Hypocholesterolemic Hypoglycemic	Caco-2	3.5 × 10^5^	17	0.5 ^d^	15–120	Inefficient intestinal transport Remarkable hydrolysis by brush border enzymes	[148]
Peptide LSW from soybean protein	ACE-inhibitory Anti-inflammatory	Caco-2	1.0 × 10^5^	21	5.0 ^d^	60	Transport of intact LSW across cell monolayer by paracellular diffusion via TJs and PepT1 pathway	[149]
Peptide lunasin and RKQLQGVN from soybean protein	Multifunctional	Caco-2	1.5 × 10^5^ ^a^	9	0.010–1.0 ^d^	60	Absorption of intact peptides across cell monolayer by paracellular diffusion	[93]
Tryptic and peptic peptides from lupin protein	Multifunctional	Caco-2	3.5 × 10^5^	18	1.0 ^c^	240	Efficient absorption of eleven tryptic and eight peptic bioactive lupin peptides	[147]
Peptide YDFYPSSTKDQQS from lupin hydrolyzate by pepsin	Hypocholesterolemic	Caco-2	3.5 × 10^5^	18	1.0 ^c^	240	Efficient absorption of peptide	[150]
Peptide fractions from *Phaseolus vulgaris* L. ecotype Controne beans	ACE-inhibitory Antioxidant α-amylase inhibitory	Caco-2	2.0 × 10^5^ ^a^	21	0.1–1.0 ^c^	120	Partial absorption of peptides across cell monolayer	[151]
Peptide YWDHNNPQIR from rapeseed protein	Antioxidant	Caco-2	1.0 × 10^5^ ^a^	21	0.025–0.25 ^d^	120	Partial absorption of peptide across cell monolayer via intracellular transcytosis Susceptibility to hydrolysis by cell peptidases	[101]
Peptides LY, RALP, and TF from rapeseed protein hydrolyzate by Alcalase^®^	ACE-inhibitory Renin inhibitory	Caco-2	1.0 × 10^5^ ^a^	21	1.0–3.0 ^d^	180	Highest absorption for peptide LY and lowest for peptide RALP Susceptibility to cell peptidases	[152]
Peptides YFCLT and GLLLPH from corn gluten	Antioxidant	Caco-2	1.0 × 10^5^ ^a^	21	4.0 ^d^	120	Absorption of intact peptides across cell monolayer via TJs-mediated paracellular diffusion and energy-dependent transcytosis Susceptibility of peptides to brush border peptidases	[141]
≤3 kDa hydrolyzate from cowpea bean protein	Hypocholesterolemic	Caco-2	5.0 × 10^4^	21	5.0 ^c^	120	Absorption of peptide MELNAVSVVHS across cell monolayer	[153]
Peptide RLSFNP from whey protein hydrolyzate with proteinases of *Lb. helveticus* LB10	ACE inhibitory	Caco-2	2.0 × 10^5^	21	1.0 ^d^	60	Absorption of intact RLSFNP and fragments F, FNP, SFNP, and RLSF Transport via paracelullar route	[154]
Peptide fractions from simulated digests of common bean milk and yogurt	Anti-inflammatory	Caco-2 clone (C2BBe1)	---	5–7	---	360	Anti-inflammatory peptides transported across the cell monolayer Yogurt samples showed higher transport efficiency than milk samples	[155]
Milk peptides LKPTPEGDL, LPYPY, IPIQY, IPI and WR	DPP-IV inhibitory	Caco-2	2.5 × 10^5^	21	1–6 ^d^	120	Low absorption capacity of peptides High susceptibility to brush border cell membrane enzymes	[123]
Milk peptide RLSFNP	ACE inhibitory	Caco-2	2.0 × 10^5^	21	1–6 ^d^	120	Transport of peptide across cells via energy-dependent transcytosis	[156]
Peptide mixture from whey protein hydrolyzed by immobilized *Lb. helveticus* proteinase	ACE inhibitory	Caco-2	2.0 × 10^5^	21	1.0 ^c^	60	Transport of peptides KA, EN, DIS, EVD, LF, AIV, and VFK across cell monolayer	[157]
Simulated digests from whey proteins	Antioxidant Immunomodulatory	Co-culture of Caco-2 (70%) and HT-29 (30%)	4 × 10^4^	10	175 ^e^	120	Bioactive peptides (ALPM, GDLE, TKIPA, VEELKPT, VGIN and AVEGPK) were transported across cell monolayer	[158]
Lactoferrin-derived peptides WQ, RWQ, and RRWQWR	Antihypertensive	Caco-2	7.5 × 10^4^	21	1.0 ^d^	120	Absorption of peptides RWQ and WQ via paracellular diffusion Susceptibility of three peptides to brush border peptidases	[145]
Peptides EAMAPK and AVPYPQ from simulated digests of Stracchino” soft cheese	Antioxidant	Caco-2	6.0 × 10^4^	14–15	0.5–4.0 ^d^	240	Absorption of peptides across cell monolayer Resistance to brush border peptidases	[142]
Peptide fraction from simulated digest of “Mozzarella di Bufala Campana DOP”	Antioxidant	Caco-2	6.0 × 10^4^	14–15	0.5–4.0 ^d^	240	Absorption of intact peptides across cell monolayer	[159]
Peptide VLPVPQK from casein hydrolyzate	ACE-inhibitory Antioxidant	Caco-2	3.0 × 10^5^ ^a^	21	0.38 ^d^	60	Partial absorption of peptide across cell monolayer via PepT1-like transporters	[160]
Peptide fractions from casein hydrolyzate by Alcalase^®^ and its simulated digest	Antioxidant	Caco-2	1.0 × 10^6^ ^b^	21	40.0 ^c^	120	Higher bioavailability for negatively charged peptides	[143]
Peptide fractions from casein hydrolyzate by Alcalase^®^	Antioxidant	Caco-2	4.0 × 10^5^	21	15.0 ^c^	120	Amino acid sequence affects peptide bioavailability Absorption via paracellular route Susceptibility to brush border peptidases	[89]
Peptide fractions from simulated digest of casein hydrolyzate by Alcalase^®^	Antioxidant	Caco-2	1.0 × 10^5^ ^b^	21	25.0 ^c^	120	High bioavailability for high hydrophobic peptide fractions	[161]
Peptides RYLGY and AYFYPEL from casein hydrolyzate by pepsin	Antihypertensive	Caco-2, HT-29-MTX and co-culture Caco-2 (75%)/HT-29-MTX (25%)	5.0 × 10^5^ ^a^	21	---	60	Absorption of intact peptides across cell monolayer Susceptibility of peptide RYLGY to cell peptidases	[162]
Simulated digests from collagen hydrolyzates by different proteases	Immunomodulatory	Caco-2	1.0 × 10^5^	21	6.0 ^c^	120	Greater transport efficiency of collagen hydrolysates due to the lower MW profile	[163]
Simulated digests from egg ovalbumin hydrolyzate (Tensiocontrol^®^)	Antihypertensive	Caco-2	1 × 10^5^ ^a^	21	0.1 ^c^	62	Protection of food matrix against bioactive peptides luminal digestion	[146]
Synthetic egg peptides IVF, YAEER, YAEERYPIL, RADHPFL, and RADHP	Antihypertensive	Caco-2	1.0 × 10^5^ ^a^	21	1.05 ^f^	25	Absorption of five egg peptides Faster transport for peptide IVF	[146]
Ovotransferrin RVPSL	Antihypertensive	Caco-2	1.0 × 10^5^ ^a^	21	5.0 ^d^	120	Partial transport of peptide across cell monolayer via TJs-mediated paracellular pathway Susceptibility to brush border peptidases	[114]
Peptides IWHHT, IWH, and IW from spent hen	ACE inhibitory	Caco-2	1.0 × 10^5^	21	5.0 ^d^	120	Partial absorption across cell monolayer Partial degradation by cell peptidases	[164]
Peptides hemorphins from simulated hemoglobin digest	Opioid	Caco-2/TC7 clone	6.0 × 10^4^	21	5.0 ^a^	60	Absoprtion of intact hemorphins across cell monolayer	[28]
Simulated digests from cooked chicken muscles	ACE inhibitory	Caco-2	2.5 × 10^5^	21	15.0 ^c^	120	Higher permeability and bioactivity for samples heated at 70 °C than at 121 °C	[165]
Peptide DLEE from Chinese dry-cured Xuanwei ham	Antioxidant	Caco-2	2.0 × 10^5^ ^b^	22	1.0–10.0 ^d^	150	Peptide absorption via paracellular transport	[144]
Peptide fraction from tilapia hydrolyzed by *V. halodenitrificans* SK1-3-7 proteinases and its simulated digests	ACE inhibitory	Caco-2	2.3 × 10^5^ ^a^	21	1.0 ^c^	360	In vitro gastrointestinal digestion enhanced the transport of hydrolyzate across cell monolayer	[166]
Peptides IQP and VEP from *Spirulina platensis*	ACE inhibitory	Caco-2	1.0 × 10^5^	21	1.0–5.0 ^d^	120	Absorption of intact peptides through cell monolayer by paracellular diffusion	[167]

^a^: density expressed as cells/well; ^b^: density expressed as cells/mL; ^c^: mg/mL; ^d^: mM; ^e^: µg protein; ^f^: µg/mL; ACE: Angiotensin converting enzyme; DPP-IV: dipeptidyl peptidase IV; MW: molecular weight; PepT1: transporter 1; TJs: tight junctions.

**Table 3:** Recent encapsulation systems developed for the delivery of food bioactive proteins/peptides.

Protein Source	Hydrolyzate/Peptide Substrate	Encapsulation Method	Outcomes	Reference
Lactoferrin	Apo-, native- and holo-lactoferrin	Alginate micro-gel particles by the aerosol technique	Protection of encapsulated apo- and native-lactoferrin from pepsin action and release in the intestinal content	[216]
Lactoferrin	Lactoferrin	Commercial microencapsulated (Progel) lactoferrin (Inferrin^TM^)	Improvement of encapsulated lactoferrin absorption in humans Beneficial effects on the human microbiome and immune system	[217]
Lactoferrin	Lactoferrin	Pectin-based colloidal delivery systems with and without chitosan coating	Retention of antimicrobial activity of systems Protection from pepsin digestion	[218]
Lactoferrin	Lactoferrin	Rapeseed phospholipid, stigmasterol, and/or HPC liposomes by thin-layer dispersion	High and moderate protection against gastric and intestinal digestion, respectively	[219]
Camel lactoferrin	Lactoferrin	Encapsulation into alginate nanocapsules	Gradual release of lactoferrin at gastrointestinal level	[220]
Bovine seroalbumin	Seroalbumin	Encapsulation into liposomes of phosphatidylcholine	Protection of encapsulated seroalbumin from pepsin action and release of protein during intestinal phase	[213]
Bovine seroalbumin	Seroalbumin	Encapsulation within xanthan gum/poly N-vinyl imidazole hydrogel	Retention of the structural integrity of protein Controlled release of seroalbumin	[221]
Seroalbumin	Seroalbumin	Encapsulation into Arabic gum-based and chitosan-based hydrogels	Slightly more efficient release of protein from the Arabic gum-based hydrogel	[222]
Azocasein	Azocasein hydrolyzate with trypsin	Encapsulation in water-in-oil-in-water double emulsions	Slow down of the release of peptides from encapsulated azocasein in the gastric phase and promotion of the peptides release in the intestinal phase	[223]
Casein	Antioxidant casein hydrolyzate by papain	Encapsulation into a maltodextrin–Arabic gum blend	Reduction of bitterness of encapsulated hydrolyzates Retention of antioxidant activity	[224]
Whey protein	Antihypertensive <3kDa fraction from hydrolyzates by proteinase from *Bacillus subtilis*	Biopolymers based on the sodium alginate matrix and filler materials (gelatin, Arabic gum, collagen)	Controlled release of ACE-inhibitory peptides from capsules subjected to simulated gastrointestinal digestion	[225]
Whey protein	Peptide fraction from hydrolyzates by papain	Encapsulation into liposomes of soybean lecithin by film hydration	Similar encapsulation efficiencies in liposomes, despite differences in the molecular weights, heterogeneities and surface hydrophobicities of whey peptides	[226]
Whey protein	Peptide fraction from hydrolyzates by papain	Encapsulation into liposomes of soybean lecithin	Lower encapsulation efficiency for anionic whey peptides than for cationic peptides	[227]
Sheep whey protein	Antioxidant and ACE-inhibitory activity of peptide fractions from ovine whey protein hydrolyzate with a *B. subtilis* proteinase	Encapsulation into liposomes of phosphatidylcholine	Retention of bioactivities in encapsulated systems	[228]
β-lactoglobulin	ACE-inhibitory peptide RLSFNP	Encapsulation into liposomes of soybean lecithin	Significant sustained release and storage capability Increase of intestinal absorption of encapsulated peptide	[156]
*Actinopyga lecanora* (stone fish) protein	Antihypertensive hydrolyzate by bromelain	Chitosan nanoencapsulation by ionotropic gelation	Higher in vivo antihypertensive efficacy in encapsulated systems	[229]
*Actinopyga lecanora* (stone fish) protein	Antihypertensive peptides from hydrolyzate with bromelain	Sodium TPP cross-linked chitosan nanoencapsulation by ionotropic gelation	Higher in vivo antihypertensive efficacy in encapsulated systems	[230]
*Onchorhynchus mykiss* (rainbow trout) skin gelatin	Antioxidant peptide fraction from hydrolyzate with Alcalase^®^	Encapsulation into phosphatidylcholine liposomes	Sustained and prolonged peptide-release behavior in a concentration-dependent manner	[231]
*Onchorhynchus mykiss* (rainbow trout) skin gelatin	Antioxidant < 30 kDa peptide fraction from hydrolyzate with Alcalase^®^	Encapsulation into chitosan-coated nanoliposomes	Sustained in vitro release of peptides Retention of antioxidant activity	[232]
*Dosidicus gigas* (giant squid) collagen	ACE-inhibitory hydrolyzate with Alcalase^®^	Encapsulation into phosphatidylcholine liposomes	Improvement of the activity of liposomes Protection during simulated gastrointestinal digestion	[233]
*Sparus aurata* (sea bream) scales	Antioxidant and ACE-inhibitory <3kDa peptide fraction from hydrolyzates by Esperase^®^ 8.0 l	Encapsulation into liposomes of soybean lecithin by film hydration	Retention of the multifunctionality of hydrolyzates during storage	[234]
Asian sea bass skin collagen	Antioxidant collagen hydrolyzates	Encapsulation into soybean phosphatidylcholine liposomes by film hydration	Retention of stability and antioxidant activity under simulated gastrointestinal digestion	[235]
*Cyprinus carpio* (carp) skin gelatin	Antioxidant hydrolyzates by Protamex^®^ enzymatic mixture	Encapsulation into furcellaran-coated microcapsules	Decrease in the in vitro and in vivo antioxidant activity of encapsulated hydrolyzates	[236]
Egg white protein	Egg white derived peptides from hydrolyzate with Alcalase^®^	Chitosan–TPP nanoencapsulation	Optimized conditions for peptides entrapment with controlled properties	[237]
Soybean 11S globulin	DPP-IV inhibitory peptide IAVPTGVA	Encapsulation into ionic self-complementary peptide hydrogels	Increase of stability under digestion conditions and bioavailability	[238]
Flaxseed protein	Antioxidant hydrolyzates (alcalase, pancreatin, trypsin, pepsin)	Maltodextrin encapsulation by spray-drying	Retention of the antioxidant activity of alcalase hydrolyzates	[239]
Flaxseed protein	Antioxidant hydrolyzates Alcalase^®^, pancreatin, trypsin)	Encapsulation into liposomes by thin-film hydration	High encapsulation efficiency Appropriate physicochemical, functional, and stability properties	[240]
Flaxseed protein	Antioxidant peptide fractions from hydrolyzates by trypsin	Maltodextrin microencapsulation by spray drying	Lower hygroscopicity, higher production yield, and better retention of antioxidant activity by spray-dried peptides	[241]
*Phaseolus lunatus* protein	Antidiabetic and antihypertensive peptides from *Phaseolus lunatus* hydrolyzate with Alcalase^®^ and Flavourzyme^®^	Maltodextrin/Arabic gum microencapsulation by spray drying	Retention of the bioactivities after simulated gastrointestinal digestion	[242]
Peanut protein	ACE-inhibitory peptide fraction from peanut protein hydrolyzate with *B. subtilis* proteinases and pepsin	Nanoliposome prepared by high pressure microfluidization	Increase of bioavailability and ACE-inhibitory activity of encapsulated peptides	[243]
Peanut protein	ACE-inhibitory peanut meal hydrolyzates with Protamex^®^ and Neutrase^®^	Encapsulation in water-in-oil-in-water multivesicular liposomes	Controlled release of bioactive peptides from liposomes Outstanding thermal stability of liposomes Retention of ACE inhibitory activity	[244]
*Citrus sinensis* (orange) seed protein	Antioxidant hydrolyzates by Alcalase^®^ and pepsin	Encapsulation into soybean and chitosan liposomes by film hydration	Protection, control of release and maintaining of the antioxidant activity of peptides	[245]
*Avena sativa* (oat) globulin	DPP-IV inhibitory hydrolyzates by trypsin	Solid lipid (triglycerides, fatty acids, steroids, and waxes) nanoparticles	Retention of stability and bioactivity of peptides under simulated gastrointestinal conditions	[246]
Brewers’ spent grain peptides	ACE-inhibitory peptides	Microencapsulation with locust bean gum, *Pyropia columbina* phycocolloids or their mixtures	Higher ACE-inhibitory activity of encapsulated peptides	[247]

ACE: Angiotensin-converting enzyme; DPP-IV: Dipeptidyl peptidase IV; HPC: hydrogenated phosphatidylcholine; TPP: tripolyphosphate.
